# Capsaicin-Sensitive Sensory Nerves and the TRPV1 Ion Channel in Cardiac Physiology and Pathologies

**DOI:** 10.3390/ijms21124472

**Published:** 2020-06-23

**Authors:** Tamara Szabados, Kamilla Gömöri, Laura Pálvölgyi, Anikó Görbe, István Baczkó, Zsuzsanna Helyes, Gábor Jancsó, Péter Ferdinandy, Péter Bencsik

**Affiliations:** 1 Cardiovascular Research Group, Department of Pharmacology and Pharmacotherapy, Faculty of Medicine, University of Szeged, H-6720 Szeged, Hungary; szabados.tamara@med.u-szeged.hu (T.S.); gomori.kamilla@med.u-szeged.hu (K.G.); gorbe.aniko@med.u-szeged.hu (A.G.); baczko.istvan@med.u-szeged.hu (I.B.); 2 Department of Physiology, Faculty of Medicine, University of Szeged, H-6720 Szeged, Hungary; palvolgyi.laura@med.u-szeged.hu (L.P.); gaborjancso@yahoo.co.uk (G.J.); 3 Department of Pharmacology and Pharmacotherapy, Semmelweis University, H-1089 Budapest, Hungary; peter.ferdinandy@pharmahungary.com; 4 Department of Pharmacology and Pharmacotherapy, Medical School & Molecular Pharmacology Research Group, Centre for Neuroscience, János Szentágothai Research Centre, University of Pécs, H-7624 Pécs, Hungary; zsuzsanna.helyes@aok.pte.hu; 5 PharmInVivo Ltd., H-7629 Pécs, Hungary; 6 Pharmahungary Group, H-6722 Szeged, Hungary

**Keywords:** acute myocardial infarction, arrhythmia, atherosclerosis, capsaicin, cardioprotection, ischemic heart disease, heart failure, transient receptor potential vanilloid type 1 (TRPV1) receptor

## Abstract

Cardiovascular diseases, including coronary artery disease, ischemic heart diseases such as acute myocardial infarction and postischemic heart failure, heart failure of other etiologies, and cardiac arrhythmias, belong to the leading causes of death. Activation of capsaicin-sensitive sensory nerves by the transient receptor potential vanilloid 1 (TRPV1) capsaicin receptor and other receptors, as well as neuropeptide mediators released from them upon stimulation, play important physiological regulatory roles. Capsaicin-sensitive sensory nerves also contribute to the development and progression of some cardiac diseases, as well as to mechanisms of endogenous stress adaptation leading to cardioprotection. In this review, we summarize the role of capsaicin-sensitive afferents and the TRPV1 ion channel in physiological and pathophysiological functions of the heart based mainly on experimental results and show their diagnostic or therapeutic potentials. Although the actions of several other channels or receptors expressed on cardiac sensory afferents and the effects of TRPV1 channel activation on different non-neural cell types in the heart are not precisely known, most data suggest that stimulation of the TRPV1-expressing sensory nerves or stimulation/overexpression of TRPV1 channels have beneficial effects in cardiac diseases.

## 1. Introduction and Background

Capsaicin is the active, pungent component of chili peppers (characterized by Scoville Heat Units, SHU, 15–16 million SHU for pure capsaicin) that is able to selectively activate and, after high dose administration, selectively defunctionalize an important subpopulation of sensory neurons with thinly myelinated Aδ or unmyelinated C fibers [1]. The actions of capsaicin are mediated through the activation of the transient receptor potential vanilloid type 1 receptor (TRPV1), formerly known as the vanilloid receptor 1 (VR1) or the capsaicin receptor [2,3], expressed predominantly but not exclusively (see below) on these chemosensitive peptidergic sensory nerves. Resiniferatoxin (RTX) is an efficient activator for TRPV1 receptors, approximately 1000 times more potent than capsaicin (with an SHU of 16 billion!), which results in activation of primary sensory neurons, [4] and repeatedly administered high doses induce ultrastructural alterations and neuropeptide depletion from these nerves similarly to capsaicin [5]. Anatomical and functional evidence indicate that capsaicin-sensitive primary afferent neurons of both spinal (thoracic dorsal root ganglia) and vagal (nodose ganglion) origin innervate the heart [6,7,8].

The incidence of cardiovascular diseases is continuously rising worldwide [9]. Ischemic heart diseases including coronary artery disease due to atherosclerotic burden, acute myocardial infarction, postischemic heart failure, and heart failure of different etiologies belong to the leading causes of death in the industrialized world [10]. In the last decades, experimental and clinical studies have identified an essential role of cardiac sensory nerves, including the capsaicin-sensitive fibers in cardiac physiology and pathologies. TRPV1 receptors expressed both on these sensory nerves and on other non-neuronal cells, as well as the mediators (mainly sensory neuropeptides) released upon their activation, play important regulatory roles in the development and/or progression of cardiovascular diseases [11]. However, despite intensive investigation in this field, data are still not coherent regarding the modulatory effects of TRPV1 and capsaicin-sensitive sensory nerves on cardiac pathologies. Therefore, in this review, we summarized data on the role of capsaicin-sensitive nerves and TRPV1 channels in cardiac physiology and pathologies and summarized their diagnostic or therapeutic potential. Available data in the literature so far are mainly obtained from preclinical experiments, since only a very limited number of clinical investigations have been performed in this field.

## 2. Capsaicin-Sensitive Sensory Nerves and TRPV1 Receptors in the Heart

**Figure 1:**
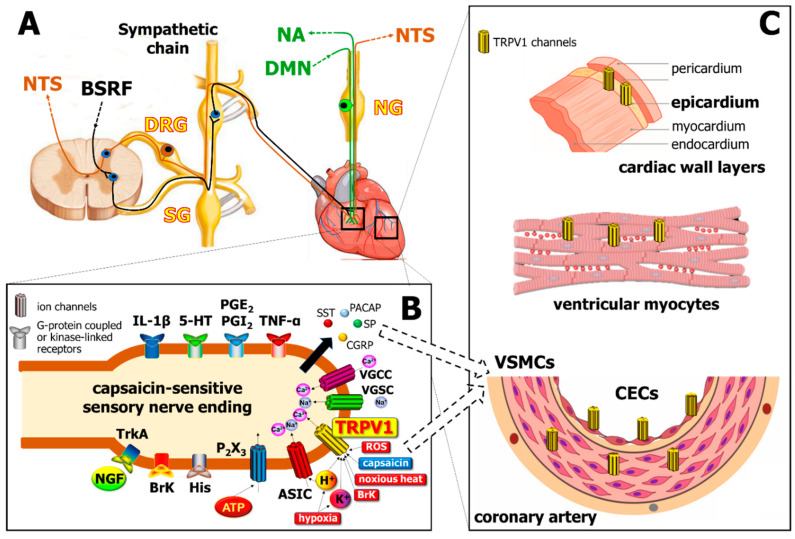
Localization of the transient receptor potential vanilloid type 1 (TRPV1) channel in different cells of the cardiovascular system. Panel (**A**): Cell bodies of primary sensory neurons innervating the heart are localized in the dorsal root ganglia (DRG) and transmit sensory signals to the nucleus tractus solitarius (NTS), as well as act as sensory efferents in the heart and the vasculature. Sensory nerves are coupled anatomically to sympathetic nerves which are derived from the brain stem reticular formation (BSRF), as well as to the vagus nerve (green), which projects afferents to the nucleus ambiguus (NA) and possesses motor efferents from dorsal motor nucleus (DMN). SG, sympathetic ganglion; NG, nodose ganglion. Panel (**B**): Several receptors co-localized with TRPV1 on the capsaicin-sensitive sensory nerve endings, which mediate a myriad of inflammatory signals including cytokines like tumor necrosis factor alpha (TNF-α) and interleukins (e.g., IL-1β), bradykinin (BrK), histamine (His), nerve growth factor (NGF; acting on Tropomyosin receptor kinase A—TrkA receptors), prostaglandins (e.g., PGE2, PGI2), serotonin (5-HT), and purine mediators (acting at P2 × 3 purinergic receptors). Noxious stimuli such as ischemia/hypoxia, increased level of reactive oxygen species (ROS), noxious heat (> 43 °C), increased levels of K+ or H^+^ activate TRPV1, and H^+^ also stimulate acid-sensitive ion channels (ASIC). Further ion channels, such as voltage-gated Na^+^ and Ca^2+^ channels (VGSC, VGCC) may also increase intracellular Na^+^ and Ca^2+^ levels, thereby inducing the release of peptide mediators, including calcitonin gene related peptide (CGRP), substance P (SP), somatostatin (SST), and pituitary adenylate cyclase-activating polypeptide (PACAP). Panel (**C**) demonstrates the localization of TRPV1 channels on different cell types of the heart (CECs, cardiac endothelial cells; VSMCs, vascular smooth muscle cells).

TRPV1 is a homotetrameric non-selective cation channel with higher selectivity for Ca^2+^ over Na^+^ [12] composed of transmembrane and cytoplasmic regions. The four identical subunits assemble around a central aqueous pore, which is located between the 5th and 6th helical segments of the transmembrane region [13,14], a stretch linking the 5th and 6th segments contains an amphipathic fragment named the P-loop, which contributes to its permeation properties [15]. TRPV1 receptors are predominantly localized on the capsaicin-sensitive peptidergic primary sensory neurons innervating the heart, but also in ventricular cardiomyocytes, vascular smooth muscle, endothelial cells, and epicardial cells [16]. TRPV1 channels are located in intracellular and mitochondrial membranes as well [17,18]. TRPV1 is activated and/or sensitized by several stimuli produced upon hypoxia, tissue injury and inflammation including noxious heat (> 43 °C) and chemical stimuli like H^+^ or K^+^, bradykinin, reactive oxygen species (ROS), and prostaglandins [19,20]. Activation of TRPV1 induces Na^+^ and Ca^2+^ influx as well as consequent membrane depolarization and the local release of several neuropeptides such as calcitonin gene-related peptide (CGRP), substance P (SP), and pituitary adenylate cyclase-activating polypeptide (PACAP) (Figure 1). These peptides contribute to vasodilation, acute phase protein activation, edema formation, and immune and inflammatory cell activation, collectively called neurogenic inflammation [21,22]. The pro-inflammatory peptides, inhibitory mediators such as somatostatin (SST), galanin, and opioid peptides, are also released from the same afferents that induce anti-edema, anti-inflammatory, analgesic, and cytoprotective actions via entry into the circulatory system, even at distant parts of the body [21,22,23,24,25,26,27]. The role of neurogenic inflammation evoked by the activation of TRPV1 channels located on the sensory nerve terminals is well-established in several vascular and dermatological pathologies (e.g., migraine, psoriasis, eczema, etc.) [28,29,30,31], but it is much less characterized in cardiovascular diseases.

The transient receptor potential ankyrin 1 (TRPA1) is a very similar ion channel that is strongly co-localized with TRPV1 on sensory nerves, vascular smooth muscle cells, and murine cardiomyocytes at the Z-discs, costameres, and intercalated discs [32]. TRPA1 is also activated by ROS, methylglyoxal, hydrogen peroxide, Ca^2+^, and prostaglandins [33,34], suggesting an interaction between TRPA1 and TRPV1.

## 3. Experimental Tools to Investigate the Function of Capsaicin-Sensitive Sensory Nerves and TRPV1 Receptors

In experimental models, it is important to distinguish the roles of neuronal and non-neuronal TRPV1 receptors. TRPV1-expressing capsaicin-sensitive primary sensory neurons are selectively defunctionalized by systemic or perineural high dose capsaicin [35,36] or RTX pre-treatment [37]. After this “chemical denervation”, peptidergic afferents cannot be activated by noxious thermal or chemical stimuli and their sensory neuropeptides are depleted [38,39]. Similarly, systemic administration of high doses of capsaicin (> 50 mg/kg) results in depletion of sensory neuropeptides from cardiac afferent nerves and the loss of sensory nerve-mediated effects [8,40,41,42].

Few studies have addressed the expression of the non-neuronal TRPV1 channel in the heart. DNA microarray and Western blot analyses have revealed the expression of TRPV1 in samples prepared from whole hearts [43] or, importantly, left ventricular cardiomyocytes [44]. There is experimental evidence that the expression of non-neuronal TRPV1 receptors, e.g., in keratinocytes, is not affected by surgical sensory denervation or pretreatment with RTX [45]. Functional studies [46,47,48] suggest that activation of non-neuronal TRPV1 receptors expressed on vascular smooth muscle cells (VSMCs) cannot be inhibited by surgical denervation or pretreatment with high-dose capsaicin abolishing vasodilator responses evoked by activation of sensory TRPV1 receptors [21,22,23,47,49,50,51,52,53]. Gene silencing or genetic knockout of TRPV1 receptors (TRPV1^−/−^) reduces or completely abrogates the expression of TRPV1 in both neuronal and non-neuronal structures, but the TRPV1-expressing sensory nerves can be activated through several other targets. Although global silencing or knockout of TRPV1 is an appropriate experimental tool to investigate the role of this specific ion channel in different pathologies, a range of adaptative compensatory mechanisms can occur during ontogenesis, and other receptors can be overexpressed to substitute its function, which is a general limitation of these models. Moreover, since TRPV1 is expressed in several cell types in the heart such as cardiomyocytes, vascular smooth muscle, and endothelial cells (see above), its absence in a global TRPV1^−/−^ animal further complicates the understanding of its exact role in cardiac pathologies. TRPV1 activity can be modulated by pharmacological agents, such as agonists (e.g., capsaicin or RTX; anandamide, etc.) and antagonists (e.g., capsazepine, SB705498, iodoresiniferatoxin, etc.), but the lack of their selectivity and often unfavorable pharmacokinetics provide several limitations. Capsazepine, the most frequently used TRPV1 antagonist, also blocks voltage-gated K^+^ and Ca^2+^ channels [54,55]. Dietary capsaicin administered in several preclinical models [56,57,58] acts on the sensory nerve terminals of the gastrointestinal system since it has low oral bioavailability and short plasma elimination half-life [59]. Although there are reports on the gastrointestinal absorption of capsaicin [60], it does not get into the systemic circulation in a sufficient amount to act directly on the TRPV1 receptors of the cardiovascular system [61]. Capsaicin is much more likely to exert anti-atherosclerotic and cardioprotective effects indirectly by reflex and/or neurohumoral mechanisms via the release of protective mediators (e.g., sensory neuropeptides such as somatostatin, CGRP, or PACAP) into the bloodstream that reach the vessels and the heart.

All these factors should be taken into consideration when evaluating the results obtained by these experimental tools and concluding on the role of TRPV1 receptors in heart diseases.

## 4. The Capsaicin-Sensitive Sensory Nerves and the TRPV1 Receptor in Cardiac Pathologies

### 4.1. Coronary Heart Disease

Atherosclerosis is now considered to be a chronic inflammatory disease, which is the primary cause of coronary artery disease (CAD). Atherosclerotic plaque rupture or erosion causes arterial thrombosis and results in acute coronary events. Treatment and regression of atherosclerosis to improve CAD is based on conventional therapy including statins, bile acid sequestrants and proprotein convertase subtilisin/kexin type 9 (PCSK9) inhibitors (to reduce circulating LDL cholesterol), beta blockers, Ca^2+^ channel blockers or diuretics (to attenuate intravascular sheer stress and intracellular Ca^2+^ burden), and platelet aggregation inhibitors (to prevent intracoronary thrombotic events) [62]. In atherosclerosis development, inflammasome signaling, including interleukin 1β and 18 signaling pathways, leads to inflammation, plaque formation, and eventually arterial occlusion [63]. Anti-inflammatory therapy against atherosclerosis is emerging, and TRPV1 receptors have been shown to participate in different immune responses [64]. In this section, we will focus on the effects of capsaicin and TRPV1 receptors on atherosclerosis and CAD, as seen in Table 1.

#### 4.1.1. In Vitro Studies

In endothelial cells (ECs), activation of TRPV1 leads to the stimulation of endothelial nitric oxide synthase (eNOS) [52,65]. Nitric oxide is responsible for smooth muscle relaxation leading to vasodilation, and it simultaneously protects endothelial cells against leukocyte adhesion. Human umbilical vein endothelial cells (HUVECs) and renal microvascular endothelial cells isolated from deoxycorticosterone-salt hypertensive mice were cultured and treated with 3–10 µM capsaicin. The results indicate that the endothelial inflammatory response is attenuated via TRPV1-mediated Ca^2+^/PI3K/Akt/eNOS/NO signaling [65]. Capsaicin (from 10 to 50 µmol) repressed ROS generation and caspase-3 activation in HUVEC cells pretreated with oxidized low-density lipoprotein (oxLDL), protected macrophage RAW 264.7 cells against foam cell formation, and induced autophagy through AMP-activated protein kinase signaling pathway in oxLDL-treated vascular smooth muscle cells (VSMCs), which suggests a protective role of TRPV1 activation against oxLDL-induced vascular dysfunctions [66,67]. TRPV1 activation by capsaicin concentration dependently increased cytosolic Ca^2+^, and significantly reduced the accumulation of lipids and cholesterol uptake in VSMCs of wild type, but not ApoE^−/−^/TRPV1^−/−^, animals. The mechanism of action for these beneficial effects of TRPV1 activation was Ca^2+^-, calcineurin- and protein kinase A-dependent via increased expression of ATP-binding cassette transporter A1 (ABCA1) and reduced expression of LDL-related protein 1 (LRP1) [57]. Lipopolysaccharide (LPS)-induced upregulation of proinflammatory cytokines including TNF-α, IL-1β, and IL-6 were markedly suppressed by the capsaicin analog dihydrocapsaicin in human monocytic THP-1 macrophage cell cultures through enhancing nuclear factor IA and inhibiting NF-κB expression [58].

#### 4.1.2. In Vivo Studies

Most in vivo studies targeting TRPV1 in animal models of atherosclerosis use dietary capsaicin treatment to achieve improvement either in atherosclerotic plaque reduction or in the accompanying inflammation. As we indicated in the above section, oral capsaicin treatment cannot directly act on cardiac or intravascular TRPV1 receptors, therefore, a remote activation should be suggested, which likely involves TRPV1 receptors localized on the capsaicin-sensitive sensory nerve terminals [68] and/or the intestinal epithelial cells [69]. The ApoE^−/−^ mouse is the most frequently used model to study atherosclerosis [70].

##### Dietary Models

Four weeks of oral treatment with evodiamine (TRPV1 agonist) induced atheroprotection in ApoE^−/−^ mice, but not in ApoE^−/−^/TRPV1^−/−^ mice, by alleviating hyperlipidemia and systemic inflammation as well as hepatic macrovesicular steatosis. However, the anti-obesity effect of evodiamine was preserved after genetic ablation of TRPV1, suggesting a TRPV1-independent mechanism [71]. Daily oral dihydrocapsaicin treatment for 12 weeks decreased proinflammatory cytokine (TNF-α, IL-1β, and IL-6) levels in the serum and in the aortic plaque of ApoE^−/−^ mice [58].

Low doses of oral capsaicin for 24 weeks reduced lipid storage and atherosclerotic lesions in the aorta isolated from ApoE^−/−^ mice, but not from ApoE^−/−^TRPV1^−/−^ mice, on a high-fat diet [57].

In a recent study, ApoE^−/−^/TRPV1^−/−^ mice were fed with either standard chow or a high-fat diet (HFD) supplemented with 0.01% capsaicin for 24 weeks. TRPV1 deficiency combined with the HFD led to coronary dysfunction, increased ROS generation, and reduced endothelial NO production. Dietary capsaicin supplementation enhanced coronary relaxation and prolonged the survival of HFD-fed ApoE^−/−^ mice, but not in ApoE^−/−^/TRPV1^−/−^ mice. Capsaicin upregulates uncoupling protein 2 expression via protein kinase A phosphorylation, thereby alleviating endothelial mitochondrial dysfunction and inhibiting mitochondrial ROS generation [56]. Guinea pigs fed by HFD for 14 weeks developed severe dyslipidemia associated with histological changes and endothelial dysfunction. Oral administration of capsaicin (2.5 mg/kg, 5 mg/kg, or 10 mg/kg once a day for 14 weeks) decreased serum levels of total cholesterol, triglycerides, LDL cholesterol, and apo B-100, and increased HDL cholesterol, as well as alleviated the plaque area, plaque area to intima ratio, and intima thickness, and also decreased the oxidative stress marker malondialdehyde and endothelin-1 levels. Therefore, dietary capsaicin supplementation may represent a promising solution for the primary prevention of CAD. However, the mechanisms between the activation of most likely the intestinal TRPV1 receptors and the vasculature to reduce atherosclerotic plaques are currently unknown.

##### Non-Pharmacological TRPV1 Activation

Activation of TRPV1 channels by capsaicin may reduce lipid storage and the formation of atherosclerotic lesions as described above. However, clinical use of capsaicin has been limited by its chronic toxicity, since long-term capsaicin consumption could damage TRPV1-expressing sensory nerves or neurons [50]. Recently, Gao et al. showed that coupling of copper sulfide (CuS) nanoparticles to antibodies targeting TRPV1 acts as a photothermal switch for TRPV1 signaling in VSMCs using near-infrared light. ApoE^−/−^ mice were fed with high-fat chow and near-infrared light was applied locally to increase the local temperature, which opened thermo-sensitive TRPV1 channels and caused Ca^2+^ influx. The influx of Ca^2+^ activated the autophagy–lysosome pathway and impeded foam cell formation in VSMCs treated with ox-LDL [72]. This alternative non-pharmacological way of TRPV1 activation may represent a novel therapeutic tool to improve atherosclerosis.

**Table 1:** Summary of the roles of TRPV1 channels in atherosclerosis.

TRPV1 Activation	Experimental Model		Beneficial Effects of TRPV1 Activation	Reference(s)
capsaicin	in vitro	HUVEC cell culture	reduced oxLDL induced ROS generation	[66]
in vivo by dietary capsaicin		VSMC of WT or TRPV1 KO mice on high-fat diet	reduced foam cell formation	[67]
dihydrocapsaicin		human monocytic THP-1 macrophage cell culture	downregulated LPS induced proinflammatory cytokines (TNF-α, IL-1β and IL-6)	[58]
capsaicin		VSMC of ApoE, TRPV1 double KO mouse	reduced lipid accumulation	[57]
CuS-TRPV1 antibody nanoparticles excited by near infrared light		VSMC of ApoE KO mouse	inhibited foam cell formation	[72]
dietary capsaicin (24 weeks)	in vivo	Apo E, TRPV1 double KO mouse on high-fat diet	reduced lipid storage and atherosclerotic lesions	[57]
daily oral gavage of dihydrocapsaicin (12 weeks)		Apo E KO mouse on high fat diet	downregulated proinflammatory cytokines (TNF-α, IL-1β and IL-6)	[58]
dietary evodiamine activation		Apo E, TRPV1 double KO mouse	alleviation of hyperlipidemia, inflammation and hepatic macrovesicular steatosis	[71]
dietary capsaicin (24 weeks)		ApoE, TRPV1 double KO mouse on high-fat diet	prolonged survival, upregulated uncoupling protein 2 expression	[56]
CuS-TRPV1 antibody nanoparticles excited by near infrared light		ApoE KO mouse	attenuated atherosclerotic lesion	[72]

#### 4.1.3. Summary

TRPV1 modulates the physiological and pathophysiological functions of vascular endothelial cells; its role in atherosclerosis is clearly demonstrated in preclinical studies. The activation of this receptor/ion channel seems to be beneficial. TRPV1 modulates the inflammatory response of endothelial cells, therefore, activation of TRPV1 should be considered as a new potential therapeutic target for the treatment of inflammatory vascular diseases, especially for atherosclerosis. However, further transcriptomics and proteomics studies would be essential to elucidate all players of the beneficial effects of TRPV1 and/or capsaicin-sensitive sensory nerve activation in atherosclerosis and CAD.

### 4.2. Myocardial Infarction and Cardioprotection

Acute myocardial infarction (AMI) is the most severe consequence of coronary atherosclerosis. The introduction of primary coronary angioplasty in the late 1970s dramatically decreased the mortality of AMI patients. However, late complications, such as postischemic heart failure, have been rising. Revelation of the nature of ischemia/reperfusion (I/R) injury led to the discovery of cardiac ischemic adaptation, such as local and remote ischemic pre- and postconditioning; however, their efficacy is affected by several comorbidities including sensory neuropathy [73,74]. Cardiac TRPV1-expressing sensory nerves and their peptide mediators (e.g., CGRP and SP) released after activation have been implicated in both local and remote cardioprotective mechanisms, but their exact roles are still debated. In the following section, we discuss the beneficial and detrimental contribution of TRPV1 and capsaicin-sensitive afferents, as well as their neuropeptides, to cardioprotection against myocardial I/R injury (see summary in Table 2).

#### 4.2.1. Acute Infarction

##### Sensory Nerve Desensitization

In an early study, a decreased cardiac level of SP was shown in myocardial ischemia; however, administration of SP attenuated ischemic myocardial damage in Langendorff-perfused hearts of rats subjected to systemic capsaicin pretreatment [75]. Two days after systemic high-dose capsaicin pretreatment, farm pigs subjected to coronary artery occlusion developed significantly higher infarct sizes, which were accompanied with decreased myocardial CGRP levels as compared to the control animals [42]. The same aggravation of myocardial damage was observed in rats 12 weeks after systemic capsaicin pretreatment at neonatal age, when infarct size was measured 6 h after coronary occlusion [76]. Interestingly, there are 2 case reports published on young male patients taking cayenne pepper pills for 5 days [77] and 3 months [78], respectively, who developed AMI without the presence of any risk factors or anamnestic data for physical or emotional stress. All the above studies and reports suggest that capsaicin-sensitive sensory nerves have a cardioprotective role against myocardial ischemic injury, at least partially due to the release of their neuropeptides including CGRP and SP.

##### TRPV1 Modulation

Capsaicin-induced TRPV1 activation in H9C2 cardiomyocytes enhanced apoptosis by increasing intracellular Ca^2+^ and mitochondrial superoxide levels, while treatment with capsazepine or TRPV1 siRNA significantly improved cell viability after hypoxia/reoxygenation (H/R) injury [79]. In contrast, TRPV1 activation has a significant cardioprotective role following myocardial I/R injury. Jiang et al. investigated the effects of TRPV1 activation on myocardial apoptosis in response to ex vivo I/R in TRPV1^−/−^ and wild type mice. To assess information about downstream signaling mechanism of TRPV1 activation by measuring phosphorylated and unphosphorylated extracellular signal-regulated protein kinase 1/2 (ERK1/2) and B-cell lymphoma 2 (Bcl-2)/Bcl-2 associated X protein (Bax) levels, they also tested I/R injury in the presence or absence of the phosphatidylinositol 3-kinase inhibitor (PI3KI), LY294002. Myocardial apoptosis and infarct size were significantly greater in TRPV1^−/−^ hearts following I/R compared with wild types and sham controls. Prior to I/R, treatment with PI3KI significantly increased myocardial apoptosis and infarct size in the wild type group, but neither have changed in TRPV1^−/−^ mice, which suggests that TRPV1 serves a protective role via the PI3K/Akt signaling pathway [80]. Moreover, Wang et al. performed a comparative study on pharmacological and genetic depletion of TRPV1 in postischemic recovery of isolated mouse hearts. Interestingly, acute pharmacological blockade of TRPV1 with capsazepine in WT mouse hearts led to more severe impairment of postischemic recovery than that observed in the TRPV1^−/−^ hearts. These results suggest that the long-term absence of TRPV1 induces compensatory mechanisms to substitute TRPV1 functions [81].

Similarly, CGRP-induced cardioprotection against AMI was demonstrated in both rodent and large animal models [42,82].

Nerve growth factor (NGF) has a key role in upregulating TRPV1 expression in peptidergic sensory neurons of adult rats [83] via the NGF-TrkA signaling. Knocking down spinal NGF had myocardial protective effect against ischemic injury similarly to the effect of capsazepine in a rat AMI model [84,85]. However, it is important to note that the sample size used in these studies were low, *n* = 3 and *n* = 6, respectively.

In contrast, the cardioprotective role of TRPV1 receptors after adenoviral delivery of the NGF gene was supported in normal and combined streptozotocin- and high fat diet-induced diabetic mouse hearts subjected to I/R injury ex vivo. Elevated levels of CGRP, but not SP, were found, which was accompanied by improved cardiac functions in both groups [86]. A recent study showed that in the heart of normal rats treated with the antidiabetic drug dipeptidyl peptidase 4 inhibitor sitagliptin orally for 2 weeks, TRPV1 and also CGRP protein levels were increased. Moreover, capsazepine co-administered with sitagliptin orally for 2 weeks abolished the cardioprotective effect of DPP-4 inhibition when rat hearts were subjected to I/R injury [87].

Morphine, a major analgesic drug used to alleviate severe pain accompanied with AMI, was shown to protect the heart against I/R injury, and this protection was partially mediated by TRPV1 receptors since TRPV1 receptor antagonists (capsazepine or P5, a peptide analgesic and TRPV1 inhibitor), prior to coronary occlusion, abrogated the cardioprotective effects of morphine [88].

#### 4.2.2. Ischemic Conditioning

The endogenous ischemic adaptation phenomena, including different forms of ischemic pre- and postconditioning, may involve capsaicin-sensitive nerve- or TRPV1-mediated cardioprotection. In ischemic preconditioning (IPC), the critical role of TRPV1 receptors was demonstrated by TRPV1 gene deletion, which abolished SP- and CGRP-mediated cardioprotection evoked by IPC [89].

##### Sensory Nerve Desensitization

Our research group has shown for the first time in the literature that capsaicin-sensitive sensory nerves are involved in preconditioning-induced cardioprotection evoked by rapid ventricular pacing. Preconditioning stimuli facilitated the release of CGRP and nitric oxide from capsaicin-sensitive nerves [40,90], which was abolished by systemic capsaicin treatment-induced sensory desensitization. Later on, a research group from the Hunan Medical University, China, demonstrated that systemic high dose (50 mg/kg) capsaicin treatment abrogates the cardioprotective effects of ischemia-, CGRP-, bradykinin-, and monophosphoryl lipid A-induced early or delayed preconditioning, respectively [91,92,93,94]. They have shown a significant decrease in the number of CGRP positive neurons, as well as decreased plasma CGRP levels, in the capsaicin-treated groups in each experimental setup.

##### TRPV1 Modulation

Remote IPC triggered by short episodes of hindlimb ischemia in rats was shown to be transferred at least partially by TRPV1 channels, since elevated left ventricular TRPV1 expression was measured after remote IPC as compared to control ischemic animals [95]. TRPV1 activation-induced CGRP release have been shown to participate in sensory nerve-mediated cardioprotection. The cardioprotective effect of limb ischemia-induced remote ischemic postconditioning was shown to be abrogated by capsazepine, the CGRP antagonist CGRP8–37, and the SP antagonist RP67580, respectively, as administered IV separately to rats [96]. Moreover, decreased myocardial TRPV1 expression was accompanied by reduced CGRP and SP release into coronary effluent after myocardial ischemia in the isolated hearts of type I diabetic rats as compared to non-diabetic ones, leading to the loss of ischemic postconditioning-induced cardioprotection and impaired myocardial function [97].

**Table 2:** Role of the capsaicin-sensitive peptidergic nerves and the TRPV1 channel in I/R injury, AMI, and cardiac ischemic conditions.

Experimental Model		TRPV1 Modulation	Effects of TRPV1 on Major Study Endpoints	Role of TRPV1 Activation	Reference
AMI + sensory desensitization	farm pig high dose capsaicin	↓	decreased infarct size and increased CGRP level	beneficial	[42]
	in vivo rat neonatal capsaicin treatment	↓	decreased infarct	beneficial	[77]
Ischemia reperfusion injury	H9C2 cell, capsazepine or TRPV1 siRNA	↓	decreased cell viability	detrimental	[79]
	ex vivo TRPV1 KO mouse	↓	decreased infarct size	beneficial	[80]
	ex vivo TRPV1 KO mouse, capsazepine	↓	improved systolic and diastolic functions	beneficial	[81]
	in vivo rat Lentivirus-mediated spinal NGF gene knockdown-induced TRPV1 downregulation	↓	increased infarct size	detrimental	[84]
	in vivo rat Lentivirus-mediated spinal NGF gene knockdown-induced TRPV1 downregulation or capsazepine induced TRPV1 inhibition	↓	increased infarct size	detrimental	[85]
	ex vivo diabetic mouse Adenovirus-mediated NGF gene delivery induced TRPV1 upregulation	↑	Elevated cardiac CGRP level and improved systolic and diastolic functions	beneficial	[86]
Sitagliptin-induced cardioprotection	ex vivo rat AMI, capsazepine and co-administration of sitagliptin	↓	capsazepine abolished infarct size limiting effects of sitagliptin	beneficial	[87]
Morphine-induced cardioprotection	in vivo rat AMI, capsazepine or P5	↓	decreased infarct size	beneficial	[88]
Early or delayed preconditioning	in vivo/ex vivo rat AMI high dose capsaicin	↓	abolished preconditioning-induced cardioprotection	beneficial	[91,92,93,94]
Pacing-induced preconditioning	ex vivo rat AMI	↓	abolished preconditioning-induced cardioprotection	beneficial	[40]
Remote ischemic preconditioning	in vivo rat AMI, RIPC induced TRPV1 upregulation	↑	decreased infarct size	beneficial	[95]
Remote ischemic postconditioning	in vivo rat AMI, capsazepine	↓	decreased infarct size	beneficial	[96]
Ischemic postconditioning	type 1 diabetic rat ex vivo AMI, capsazepine	↓	abolished IPost-induced cardioprotection	beneficial	[97]

#### 4.2.3. Summary

These experimental data show predominantly protective roles of cardiac capsaicin-sensitive afferents and sensory TRPV1 receptors in myocardial protection through the release of sensory neuropeptides. However, involvement of TRPV1 receptors expressed by cardiomyocytes [43,98] and endothelial cells has not been investigated. Although several studies have been performed to investigate the alterations in proteomics or transcriptomics, including microRNA (miRNA)-omics, related to myocardial I/R injury or cardioprotective maneuvers like ischemic conditionings, surprisingly, involvement of TRPV1 or capsaicin-sensitive sensory nerves in such studies is still an unmet need.

### 4.3. Heart Failure

Heart failure (HF) is a complex clinical syndrome resulting from the decreased function of the right, left, or both ventricles. The symptoms derive from an inadequate cardiac output, since the failing heart is unable to keep up with the demands [99]. Three main phenotypes describe HF based on the measurement of the left ventricular ejection fraction (EF). HF with reduced ejection fraction (HFrEF), HF with preserved EF (HFpEF), and HF with mid-range EF (HFmrEF) [100]. As we indicated in the previous section, increased survival rate after AMI due to recanalization of the coronaries via percutaneous coronary intervention constitutes one of the major sources for HFrEF patients. However, since causal therapy is not available against HFrEF, there is an utmost need to discover novel pathways and targets to improve cardiac function and prolong life expectancy of HF patients.

The roles of capsaicin-sensitive sensory nerves and/or TRPV1 receptors in the development or progression of HF have been investigated intensively in the last 2 decades. Various animal models of HF including post-infarction-, high salt diet/hypertension-, pressure overload-, or toxic cardiac injury (e.g., doxorubicin)-induced models showed important roles of TRPV1 in HF. However, its exact function in the pathophysiological mechanisms, as well as its appraisal, still remains unclear (see Table 3 for details).

#### 4.3.1. Sensory Desensitization in HF Studies

In rats, neonatal capsaicin treatment ablating the capsaicin-sensitive fibers resulted in an enhanced pressor response to exercise comparable to post-infarction HF, and altered the activity and sensitivity of the sensory fibers in HF [101]. Our research group has previously shown that capsaicin-induced sensory desensitization causes an impaired myocardial relaxation characterized by increased LV end-diastolic pressure [102]. We have also demonstrated that systemic capsaicin treatment can aggravate myocardial dysfunction induced by adriamycin-evoked congestive cardiomyopathy [8]. However, cardiac effects of systemic sensory desensitization have not yet been investigated in the presence of other etiologies of HF. Recently, we have found an increased end-diastolic diameter, and reduced stroke volume, but maintained EF and FS, after systemic capsaicin-induced sensory desensitization in rats [103]. Disturbed relaxation was due to reduced basal cardiac NO, superoxide, and peroxynitrite (ONOO^−^) formation, and thereby impairment of the physiological S-nitrosylation level of the sarco-endoplasmic reticulum Ca^2+^ ATPase 2a (SERCA2a) [104]. Moreover, we have also investigated the effects of systemic capsaicin-induced sensory neuropathy on cardiac miRNA transcriptomics and shown an altered miRNA expression pattern. We further characterized the altered cardiac miRNA profile by using unbiased bioinformatic target prediction and identified as well as evaluated novel target genes, which can be responsible for the development of diastolic dysfunction (for details, see reference [103]). This model of sensory neuropathy due to capsaicin-induced sensory desensitization provides an experimental HFpEF model. The role of TRPV1 in the development of HFpEF has not been investigated so far. In contrast with these results, high dose RTX treatment resulting in complete epicardial ablation of TRPV1-expressing nerves on the surface of the rat heart led to a significantly improved HF indicated by LV systolic and diastolic diameters and volumes, but infarct size was not affected [105]. In addition to the latter study, intrathecal RTX treatment in rats ablating the TRPV1+ afferents in the spinal cord protected the heart against TAC-induced interstitial fibrosis and attenuated LV hypertrophy. Eight weeks after the surgery, the rats had similar systolic parameters, including EF and FS, to sham rats [106].

#### 4.3.2. Studies on Pharmacological and Genetic Ablation of TRPV1

##### Post-Infarction HF

Two research groups [101,107] investigated the relationships between exercise pressor reflex (EPR) and TRPV1 in HF. Both studies showed that mRNA expression of TRPV1 was significantly downregulated in rats with coronary ligation-induced dilative cardiomyopathy [101,107]. Wang et al. showed that group III afferents (predominantly mechanically sensitive) are sensitized and group IV afferents (predominantly metabolically sensitive) are blunted in rats with post-infarction HF [107]. In TRPV1^−/−^ mice, TGF-β and Smad2 expression were upregulated, and the infarct size was significantly greater 7 days after permanent ligation of the left coronary artery as compared to wild types [108]. Moreover, in the hearts of end-stage HF patients, TRPV1 mRNA expression showed an increasing tendency as compared to healthy controls [109].

##### Chronic Hypertension-Induced HF

An early study using Dahl salt-sensitive and salt-resistant rats subjected to low- and high-salt diets for 3 weeks was the first to show that TRPV1 protein expressions in mesenteric arteries and kidney samples are upregulated due to increased salt intake and the overexpression of TRPV1 prevents the development of salt-induced hypertension only in salt-resistant animals [110]. Lang et al. [111] investigated the effect of daily capsaicin intake for 24 weeks in high-salt diet-induced cardiac hypertrophy in TRPV1^−/−^ mice. They showed that after chronic dietary capsaicin intake, mitochondrial Complex I activity, ATP production, and citrate synthase activity were significantly increased, and mitochondrial sirtuin 3 was upregulated in WT mice on high-salt diet through a TRPV1-dependent manner since these alterations were not manifested in TRPV1^−/−^ mice [111].

##### Pressure Overload-Induced HF

In rodent models of transverse aortic constriction (TAC), which is employed to induce cardiac pressure overload, thereby cardiac hypertrophy and HF, the role of TRPV1 has intensively been investigated in the last decade. It has been disclosed that TAC-induced pressure overload upregulates cardiac TRPV1 expression [112]. Mice lacking functional TRPV1 developed significantly reduced cardiac hypertrophy induced by TAC measured over time until 8 weeks after TAC [113]. However, 4 weeks after TAC-induced ventricular hypertrophy, TRPV1^−/−^ mice had significantly decreased LV ejection fraction (EF) and fractional shortening (FS) as well as significantly increased secretion of pro-inflammatory cytokines, TNFα and IL-6 [112]. In a suprarenal aortic banding-induced HF model, 10 weeks of 0.01% dietary capsaicin intake significantly attenuated the hypertrophic response in WT mice, but not in TRPV1^−/−^ mice [114].

##### HF Models of Toxic Cardiac Injury

Indirect evidence supports the protective role of TRPV1 channels against anthracycline (i.e., doxorubicin)-induced HF in rat model [115,116,117]. Eugenol, as an active component of clove oil, is an antioxidant as well as a TRPV1 activator and has previously been shown to possess cardioprotective effects [118]. Eugenol administered orally for five days for doxorubicin-treated rats improved cardiac function, which was abolished by capsazepine [117]. A recent study was consistent with the above findings, where the authors treated wild-type mice and mitochondrial aldehyde dehydrogenase 2-overexpressing transgenic (ALDH2TG) mice with doxorubicin to induce congestive heart failure characterized by left ventricular dysfunction and chamber dilation [116]. ALDH2 was previously found to be cardioprotective against ischemia-reperfusion injury and alcoholic cardiomyopathy due to detoxification of toxic reactive aldehydes [119]. SA13353, a TRPV1 agonist, was administered to wild type mice, and parallelly, capsazepine was administered to ALDH2TG mice to assess the involvement of TRPV1 receptors in the protection of ALDH2 against doxorubicin-induced cardiac dysfunction. Treatment of wild type mice with SA13353 led to improved cardiac function, while capsazepine administered to ALDH2TG mice abrogated increased contractility evoked by ALDH2 overexpression, respectively, which indicates that cardioprotection induced by ALDH2 overexpression against doxorubicin-induced heart failure is mediated at least partially by TRPV1. The latter finding was confirmed at the cellular level in cultured cardiac myocytes [116].

It is interesting to note that the role of TRPV1 in cardiac dysfunction associated with sepsis and endotoxemia is also under investigation. Chen et al. [120] showed the cardioprotective role of TRPV1 in a mouse model of LPS-induced endotoxemia in TRPV1^−/−^ mice. Low-dose LPS did not cause any cardiac dysfunction in wild type mice, but led to a significant reduction in EF, FS, and fraction area change% (FAC), and caused bradycardia in TRPV1^−/−^ mice. Results after the TRPV1 antagonist AMG-9810 treatment were similar to TRPV1 deletion, suggesting that TRPV1 may have a key role in cardioprotection against endotoxin-induced cardiac dysfunction [120].

**Table 3:** Summary for the role of TRPV1 channels in HF.

	Treatment to Modulate TRPV1	TRPV1 up- or Downmodulation	Experimental Model	Effect of Treatment on Major Study Endpoints	Role of TRPV1 Activation on Cardiac Remodeling	Reference(s)
Capsaicin-sensitive sensory nerve desensitization	neonatal capsaicin treatment	↓	neonatal capsaicin treatment, dilated cardiomyopathy (DCM) and control rat	enhanced EPR compared to control	detrimental	[101]
	epicardial TRPV1 ablation by high dose RTX	epicardial ↓	post-MI-induced HF with RTX treatment in rat	improved cardiac compliance	detrimental	[105]
	intrathecal RTX treatment	spinal cord ↓	transverse aortic constriction (TAC)-induced HF	improved cardiac function	detrimental	[106]
	sc. capsaicin treatment for 3 days at increasing doses	↓	sensory neuropathy-induced HFpEF	impaired myocardial relaxation	beneficial	[103,104]
Pharmacological or genetic modulation of the TRPV1 receptor	genetic deletion	↓	7 days post-MI mouse	increased infarct size	beneficial	[108]
	genetic deletion and dietary capsaicin for 24 weeks	↑	high-salt diet-induced cardiac hypertrophy, mouse	improved mitochondrial function	beneficial	[111]
	TRPV1 gene disruption	↓	TAC-induced HF	reduced cardiac hypertrophy	detrimental	[113]
	genetic deletion	↓	TAC-induced HF	decreased cardiac function and increased TNFα and IL-6	beneficial	[112]
	genetic deletion and dietary capsaicin for 10 weeks	↑	TAC-induced HF	attenuated hypertrophy in WT	beneficial	[114]
	TRPV1 activation by eugenol, capsazepine	↑	acute doxorubicin cardiotoxicity	improved cardiac function	beneficial	[117]
	SA13353 TRPV1 agonist, and capsazepine	↑	doxorubicin-induced HF, ALDH2 transgene mouse	improved cardiac function	beneficial	[116]
	genetic deletion, AMG-9810 TRPV1 antagonist	↓	LPS-induced endotoxemia, mouse	cardiac dysfunction	beneficial	[120]

#### 4.3.3. Summary

The vast majority of the studies demonstrate cardioprotective roles of the capsaicin-sensitive nerves and TRPV1 receptors on cardiac functions independently of the etiology of HF. Although some studies reported on beneficial effects after the depletion of TRPV1-expressing nerves/neurons, these studies used either extracardiac desensitization [106] or superficial treatment (i.e., painting the epicardium with a brush immersed into RTX solution) [105] to deplete epicardial TRPV1 receptors.

### 4.4. Arrhythmias and Electrophysiology

Cardiac arrhythmias can be classified as ventricular and supraventricular arrhythmias. Excessive sympathetic activity contributes to structural and electrical remodeling of the myocardium in different cardiac pathologies including AMI and HF and increases arrhythmia susceptibility, the incidence of severe ventricular arrhythmias, and sudden cardiac death [121,122]. Approximately half of all mortality in chronic HF is due to sudden cardiac death, a significant proportion of which is caused by ventricular tachyarrhythmias [123]. Reperfusion of previously ischemic myocardium (e.g., during recanalization following AMI) can also lead to the development of severe ventricular arrhythmias both in preclinical and clinical settings [124,125]. Only a few studies investigated the effects of TRPV1 modulators on cardiac electrophysiology and arrhythmias in experimental models.

#### 4.4.1. Ventricular Arrhythmias

##### TRPV1-Independent Cardiac Actions of Capsaicin and RTX

The TRPV1-independent actions of capsaicin and RTX on cardiac K^+^ channels were demonstrated in a somewhat contradictory study. Capsaicin prolonged single cell APD and inhibited the transient outward K^+^ current (I_to_), a voltage dependent non-inactivating outward current (I_K_), and the inward rectifier K^+^ current (I_K1_) currents in isolated adult rat ventricular cardiac myocytes [126]. Interestingly, the capsaicin analogue zingerone exhibited similar effects, but RTX did not influence any of these currents [126]. To resolve this contradiction, the author clarified the differential effects of capsaicin and RTX on K^+^ channels by differential structural requirements for K^+^ channel blockade in the heart as compared to those required to produce neuroexcitatory effects. This finding also indicates that the vanillyl moiety of capsaicin and zingerone is essential for blocking cardiac K^+^ channels. In guinea pig ventricular papillary muscle preparations, capsaicin significantly shortened the action potential duration (APD) and did not influence the maximum rate of depolarization, suggesting no voltage-gated Na^+^ channel (I_Na_) involvement. The authors speculated that AP shortening and antiarrhythmic effect of capsaicin was due to the blockade of voltage-gated Ca^2+^ channels (I_CaL_), although no ionic currents were measured [127].

##### Sensory Desensitization Studies on Ventricular Arrhythmias

In a rat model with HF induced by 4-week coronary ligation, intrathecal RTX pre-treatment significantly reduced dorsal horn TRPV1 expression and reduced cardiac sympathetic nerve overactivation, which reversed epicardial monophasic APD prolongation and decreased action potential alternans and ventricular arrhythmia development evoked by programmed electrical stimulation [128]. In a porcine model of AMI induced by permanent obliteration of the left anterior coronary artery, capsaicin-sensitive nerve defunctionalization evoked by epicardial RTX administration prevented ventricular tachycardia and fibrillation induced by programmed electrical stimulation [129]. In RTX-treated AMI pigs, no changes were observed in cardiomyocyte action potential parameters, ion channel expression, or calcium transients at the infarct border zone, while electrical heterogeneity, fibrosis, and altered distribution of connexin 43 was attenuated compared to untreated AMI animals [129].

##### Studies on Pharmacological and Genetic Ablation of TRPV1

In an early study, the incidence of ventricular tachycardia and fibrillation induced by either global or regional myocardial ischemia was reduced in isolated guinea pig and rat hearts, respectively, when 30 µM capsaicin was administered to Krebs-Henseleit solution prior to ischemia [127]. In rats subjected to 30 min of myocardial ischemia followed by 120 min reperfusion, TRPV1 mRNA was upregulated in the dorsal root ganglia (DRG) neurons [130]. Arrhythmia score along with infarct size was significantly reduced when TRPV1 upregulation was suppressed by lentiviral TRPV1 gene silencing in the DRG. Similar antiarrhythmic effects were observed after exposure to the TRPV1 antagonist iodoresiniferatoxin [130]. Recent studies suggest that chronically elevated cardiac sympathetic excitation driven by persistent TRPV1 afferent signaling in pig and rat models, respectively, is associated with cardiac remodeling and arrhythmia generation [128,129]. Importantly, TRPV1 has also been identified as the regulated molecular component of the atrial natriuretic signaling pathway and as a potential molecular target in HF and cardiac hypertrophy induced by TAC in mice. Reduction of cardiac structural and electrical remodeling in HF by beneficial modulation of the atrial natriuretic signaling pathway may markedly decrease both arrhythmia substrate and triggered activity [131].

#### 4.4.2. Supraventricular Arrhythmias

Atrial fibrillation (AF) is the most common chronic arrhythmia with multiple underlying mechanisms, particularly heart failure, where AF prevalence is approximately up to 40% at time of hospitalization [132]. The autonomic nervous system critically influences atrial electrophysiology, and its dysfunction is considered to be a major factor in the initiation and maintenance of AF [133]. However, little is known about a possible link between capsaicin-sensitive sensory nerves/TRPV1 function/dysfunction and AF. Obstructive sleep apnea is associated with the development of AF via its effects mediated by the cardiac autonomic nervous system [134]. In a very recent study, the injection of RTX into the cardiac ganglionated plexi (GP) decreased nerve and stellate ganglion activity, abolished sleep apnea, induced the shortening of the effective refractory period (ERP), and completely inhibited AF induction in a dog model of sleep apnea [135]. In patients with non-valvular AF, a marked increase in TRPV1 expression was found in leukocytes compared to control patients without AF [136], suggesting enhanced systemic inflammatory response status and oxidative stress in patients with non-valvular AF, both identified as important contributors to AF induction and maintenance [137].

#### 4.4.3. Summary

In summary, these relatively few studies investigating the cardiac electrophysiological effects of TRPV1 and its modulators suggest that TRPV1 plays a significant role in arrhythmogenic cardiac electrical and structural remodeling associated with AMI, HF, and AF. Importantly, different TRPV1 modulators have been shown to possess different direct cardiac ion channel effects, which need to be further explored in order to dissect the role of TRPV1 in cardiac electrophysiology and arrhythmias, preferably in animal models that are relevant to human diseases [138,139].

### 4.5. Congenital Heart Diseases

Congenital heart diseases (CHD) are among the most common birth defects [140]. Malformations of the developing heart during the intrauterine life are related to both genetic and environmental factors. Ebstein’s anomaly, Fallot tetralogy, and Wolff-Parkinson-White syndrome did not show connection with the TRPV1 receptor. On the other hand, environmental factors, like heat (e.g., maternal fever during the first trimester) showed correlation to CHD [141]. Based on these phenomena Hutson et al. designed a proof-of-concept study with chick embryos and reported that heat-activated TRPV1 and TRPV4 channels were present in neural crest cells during critical window of heart development and they induced fever-associated defects. These data provided a mechanism for hyperthermia-activated TRPV1 ion channel activation resulting in congenital heart defects. Blocking TRPV1 during heat exposure mitigated most of the hyperthermia-induced heart malformations [142]. This special field of cardiology requires more intensive investigations to reveal the role of TRPV channels in heart development.

## 5. Conclusions and Future Perspectives

This review summarizes the data about the role of the cardiac capsaicin-sensitive sensory nerves and the TRPV1 receptor in cardiac pathologies, including coronary heart disease, myocardial infarction, heart failure, and arrhythmias. Sensory neuronal TRPV1 activation seems to play a crucial role in reducing atherosclerosis and confers significant cardioprotective effects against ischemic damage. However, although little is known about TRPV1 functions in cardiac electrophysiology, it is likely to play a role in supraventricular and ventricular arrhythmogenesis. Although most data suggest that stimulation of the TRPV1-expressing sensory nerves or the TRPV1 channel might have beneficial effects in cardiac diseases, this conclusion should be cautious. The interactions of other ion channels and receptors expressed on the capsaicin-sensitive sensory afferents with TRPV1, as well as the actions of TRPV1 channel activation on non-neuronal cell types in the heart, are not precisely known. Therefore, future studies should target cell type-specific TRPV1 receptors, use more selective pharmacological interventions, and analyze clinical samples to elucidate the role and mechanism of action of sensory and non-neuronal TRPV1 signaling, as well as to determine its value for potential novel therapies. Multi-omics studies, including transcriptomics, microRNAomics, and proteomics, as well as bioinformatic synthesis of the data, are required to discover the signaling of the TRPV1 channel and capsaicin-sensitive sensory nerves in cardiac pathologies in an unbiased manner. Our previous omics studies [43,103] on cardiac capsaicin-sensitive sensory nerves revealed some novel potential targets, which should be further validated in vivo. However, we investigated the effects of sensory desensitization alone on cardiac function and metabolism. In future omics studies, sensory desensitization or pharmacological/genetic ablation of TRPV1 should be combined with conventional cardiac pathology models to elucidate further targets, which may contribute to the pathomechanism of ischemic heart diseases, heart failure, or cardiac arrhythmias due to the lack/dysfunction of sensory nerves/TRPV1 channels. Moreover, large animal models of sensory desensitization/TRPV1 ablation combined with cardiac diseases are essential to be developed to gain translatable findings, which can be used to develop adaptable therapies for humans.

## Abbreviations

5-HT	Serotonin
ABCA1	ATP-binding cassette transporter A1
ALDH2	Aldehyde dehydrogenase 2
AMI	Acute myocardial infarction
APD	Action potential duration
ASIC	Acid-sensitive ion channels
BAX	B-cell lymphoma 2 associated X protein
BCL2	B-cell lymphoma 2
BrK	Bradykinin
BSRF	Brain stem reticular formation
CAD	Coronary artery disease
CEC	Cardiac endothelial cell
CGRP	Calcitonin gene-related peptide
CHD	Congenital heart diseases
DCM	Dilated cardiomyopathy
DMN	Dorsal motor nucleus
**DOAJ**	**Directory of open access journals**
DRG	Dorsal root ganglia
EC	Endothelial cell
EF	Ejection fraction
eNOS	Endothelial nitric oxide synthase
EPR	Exercise pressor reflex
ERK1/2	Extracellular signal-regulated protein kinase 1/2
ERP	Effective refractory period
FS	Fractional shortening
H/R	Hypoxia/reoxygenation
HF	Heart failure
HFD	High-fat diet
HFmrEF	Heart failure with mid-range ejection fraction
HFpEF	Heart failure with preserved ejection fraction
HFrEF	Heart failure with reduced ejection fraction
HUVEC	Human umbilical vein endothelial cell
I/R	Ischemia/reperfusion
IPC	Ischemic preconditioning
LRP1	LDL-related protein 1
**MDPI**	**Multidisciplinary Digital Publishing Institute**
NA	Nucleus ambiguus
NG	Nodose ganglion
NGF	Nerve growth factor
NTS	Nucleus tractus solitarius
ONOO^-^	Peroxynitrite
PACAP	Pituitary adenylate cyclase-activating polypeptide
PCSK9	Proprotein convertase subtilisin/kexin type 9
PI3KI	Phosphatidylinositol 3-kinase inhibitor
ROS	Reactive oxygen species
RTX	Resiniferatoxin
SERCA2a	Sarco-endoplasmic reticulum Ca^2+^ ATPase 2a
SG	Sympathetic ganglion
SHU	Scoville Heat Unit
SP	Substance P
SST	Somatostatin
TAC	Transverse aortic constriction
TNF	Tumor necrosis factor alpha
TrkA	Tropomyosin receptor kinase A
TRPV1	Transient receptor potential vanilloid type 1
VGCC	Voltage-gated Ca^2+^ channel
VGSC	Voltage-gated Na^+^ channel
VMSC	Vascular smooth muscle cell
